# Inequalities in the Quality and Safety of Post‐Diagnostic Primary Care for People With Dementia: A Scoping Review

**DOI:** 10.1002/gps.70035

**Published:** 2024-12-23

**Authors:** Charlotte Morris, Roukia Techache, Katie Davies, Tom Blakeman, Evangelos Kontopantelis, Darren M. Ashcroft, Dame Louise Robinson

**Affiliations:** ^1^ NIHR School for Primary Care Research Department of Primary Care and Health Services Research The University of Manchester Manchester UK; ^2^ National Institute for Health and Care Research Greater Manchester Patient Safety Research Collaboration School of Health Sciences Faculty of Biology, Medicine and Health University of Manchester Manchester UK; ^3^ Foundation Programme Doctor Salford Royal Hospital Salford UK; ^4^ National Institute for Health Research School for Primary Care Research Centre for Primary Care Manchester Academic Health Science Centre University of Manchester Manchester UK; ^5^ The Healthy Ageing Research Group The University of Manchester Manchester UK; ^6^ Division of Informatics, Imaging and Data Sciences The University of Manchester Manchester UK; ^7^ Division of Pharmacy & Optometry School of Health Sciences Faculty of Biology, Medicine and Health University of Manchester Manchester UK; ^8^ Population Health Science Institute Faculty of Medical Science Newcastle University Newcastle‐upon‐Tyne UK

**Keywords:** dementia, health inequality, patient safety, primary care, quality

## Abstract

**Introduction:**

International guidelines make recommendations for the delivery of safe, high‐quality primary care for people with dementia including prescribing, personalised care planning and regular holistic reviews. It is unclear how the quality and safety of this healthcare varies with socio‐economic factors.

**Objective:**

This scoping review aimed to understand the depth and breadth of existing evidence exploring socio‐economic variation in the quality and safety of primary care for people with dementia.

**Methods:**

Prescribing and care planning indicators of high‐quality, safe primary care were defined from guidance. Composite and proxy markers of socio‐economic status (SES) were defined. EMBASE, MEDLINE, PsychInfo, The Cochrane Database of Systematic Reviews, worldcat.org and clinicaltrial.gov databases were searched. Studies in English, on human participants from 2006 onwards were eligible. Narrative synthesis was conducted. Studies explored how one or more selected indicators (anti‐dementia medication and anti‐psychotic prescribing, potentially inappropriate prescribing (PIP), medication review, dementia review or care planning) varied with a recognised marker of SES in people with dementia.

**Results:**

Searches identified 1980 studies after removing duplicates. 385 full texts were reviewed, with 53 eligible for inclusion (51 quantitative, 2 reviews). Most identified studies explored prescribing processes (50 quantitative, 2 reviews), with 2 exploring annual review.

There was evidence of substantial disparity in quality and safety indicators in studies exploring prescribing; 20/29 (69%) of studies exploring anti‐dementia medication prescribing found those with markers of lower SES were significantly less likely to receive these. 16/28 studies exploring PIP/Anti‐psychotics found significant disparities in safe prescribing for those with markers of lower SES. Neither study exploring annual reviews found any significant differences across SES.

**Conclusion:**

We found evidence of disparity in the quality and safety of post‐diagnostic primary care for people with dementia based on SES, particularly for a range of prescribing indicators. Further work exploring inequalities in care planning and reviews for people with dementia is needed to understand existing inequalities in the quality and safety of primary care for people with dementia.

**Summary**

- What's already known about this topic?
  ◦ Studies have explored how recommended indicators of high‐quality, safe primary care for people with dementia vary with socio‐economic factors. These studies have explored different indicators and heterogenous measures of SES.
- What does this study add?
  ◦ To our knowledge, this is the first scoping review to explore and synthesise the breadth of existing literature exploring how the quality and safety of primary care for people with dementia varies with different markers of SES. The study identified which indicators have been well explored, and which require further investigation.
- How might the study affect research, practice, and policy?
  ◦ The study identifies priority areas for future work exploring how the quality and safety of primary care varies for people with dementia. Clinicians should be aware of potential disparities in access to high quality safe care, and the possible reasons for these. Understanding existing inequalities is essential for research, practice, and policy.

## 1 Introduction

Dementia is a global health priority [1, 2]. Cases are projected to increase to 152.8 million people by 2050 [3]. Dementia is a progressive condition, which can leave people reliant on care from others [4, 5]. Primary care services are often the main healthcare provider for people with dementia [6]. This healthcare needs to be safe, high‐quality, and equitable.

Projected increases in dementia prevalence suggest low‐ and middle‐income countries (LMICs) will be most affected [3, 7] but there is evidence health inequality is vast and growing within countries [8]. This study focuses on inequalities in primary care provision within and across countries. There is evidence that people from poorer backgrounds within high‐income countries (HICs) develop dementia at younger ages and die from dementia sooner than their more affluent counterparts [9, 10]. Dementia risk‐factors cluster around deprivation [11], including smoking, obesity, and lower educational attainment. This suggests even in HICs there will be disproportionate increases in dementia incidence in people living in deprivation. With the advent of new, expensive treatments for dementia, it is important that these medications are available to all who need them, and that provision is based on clinical need. Until existing inequalities are understood, it will be difficult to design models of care which promote equity through local or national policies.

International guidelines make recommendations for the delivery of safe, high‐quality post‐diagnostic primary care for people with dementia [2, 4, 9, 12, 13, 14, 15, 16, 17, 18]. People with dementia are at high‐risk of iatrogenic harm through sub‐optimal prescribing, or inadequate medication review; for example, anti‐psychotic prescribing is associated with multiple severe, life threatening harms for people with dementia [19]. Anti‐cholinergic medications are associated with worsening cognition, stroke [20], and adverse functional outcomes [21]. To achieve high‐quality, safe care, regular person‐centred reviews are recommended. These aim to co‐ordinate care, review medications, discuss preferences for care, and make appropriate referrals. Previous work has shown the quality of these reviews is highly variable [6, 22], but has not explored variation with SES.

It is hypothesised people with dementia with markers of lower SES are less likely to receive high‐quality, safe primary care compared to those with higher SES. A recent systematic review explored inequalities in care pathways for people with dementia [23]. The review did not explore variation of guideline consistent primary healthcare with SES, focussing instead on care pathways including diagnosis, care transitions, mortality, and limited prescribing indicators. The review only included studies exploring electronic health record or cohort data. This highlighted a gap in the literature for a scoping review exploring the depth and breadth of literature relating to how the quality and safety of primary care varies with SES.

This scoping review had two aims:

1. to map existing quantitative studies exploring variation in the quality and safety of primary care for people with dementia with SES, analysing knowledge gaps.
2. to conduct a narrative synthesis of these studies

## 2 Method

A protocol was designed a priori; the review was conducted in accordance with the ‘Prisma‐SCR’ Checklist [24]. Scoping review methodology was used because the study's aim was to identify relevant literature and analyse knowledge gaps [25]. This paper presents a scoping review of quantitative studies exploring prescribing and care planning indicators of quality and safety of primary care for people with dementia.

### 2.1 Defining High‐Quality, Safe Primary Care for People With Dementia

High‐quality healthcare must be effective, safe, person‐centred, and equitable [26, 27]. There are multiple sets of international guidance for providing high‐quality, safe primary care for people with dementia [2, 4, 12, 13, 14, 15, 16, 17, 18, 28]. The most frequently used recommendations in the UK were developed by National Institute for Health and Care Excellence (NICE) [4]. Table 1 shows recommended care processes, synthesised from existing English language international guidance documents [2, 4, 12, 14, 18].

**TABLE 1:** Recommended primary care processes for people with dementia.

	Guideline indicators of quality/safety	UK Guidance recommending	International guidance recommending
**Care planning**	Personalised dementia care planning	NICE [4], NCCMH Dementia care pathway [12], NHS Good Care Planning [13], SIGN [15]	New Zealand framework [17]
			Australian clinical practice guidance [16]
			World Alzheimer's report [2]
	Annual review^a^	NICE [4], NCCMH Dementia care pathway [12], NHS Good Care Planning [13], SIGN [15] Quality and Outcomes Framework [14]	New Zealand framework [17] (quarterly, not annual)
			World Alzheimer's report [2]
	Continuity of care	NICE [4], NCCMH Dementia care pathway [12], NHS Good Care Planning [13], SIGN [15]	New Zealand framework [17]
			Australian clinical practice guidance [16]
			World Alzheimer's report [2]
**Prescribing**	Minimisation of PIP	NICE [4], NCCMH Dementia care pathway [12], NHS Good Care Planning [13], SIGN [15]	New Zealand framework [17]
			Australian clinical practice guidance [16]
			World Alzheimer's report [2]
	Avoidance of anti‐psychotic prescription and review at 6 weeks	NICE [4], NCCMH Dementia care pathway [12], NHS Good Care Planning [13], SIGN [15]	Australian clinical practice guidance [16]
	Anti‐dementia medication prescribing	NICE [4], NCCMH Dementia care pathway [12], NHS Good Care Planning [13], SIGN [15]	New Zealand framework [17]
			Australian clinical practice guidance [16]
			World Alzheimer's report [2]
	Medication review	NICE [4], NCCMH Dementia care pathway [12], NHS Good Care Planning [13], SIGN [15]	New Zealand framework [17]
			Australian clinical practice guidance [16] (at diagnosis)
	Polypharmacy avoidance	NICE [4], NHS Good Care Planning [13], SIGN [15]	New Zealand framework [17]
**End of life care**	Advance care planning	NICE [4], NCCMH Dementia care pathway [12], NHS Good Care Planning [13], SIGN [15]	New Zealand framework [17]
			Australian clinical practice guidance [16]
			World Alzheimer's report [2]
			European Association for palliative Care recommendations [28]
	Access to needs‐based community palliative care and appropriate referral to specialist services	NICE, NCCMH Dementia care pathway [12], NHS Good Care Planning [13], SIGN [15]	New Zealand framework [17]
			Australian clinical practice guidance [16]
			World Alzheimer's report [2]
			European Association for palliative Care recommendations [28]
	Death at preferred place of death	NICE [4], NCCMH Dementia care pathway [12], NHS Good Care Planning [13], SIGN [15]	New Zealand framework [17]
			Australian clinical practice guidance [16]
			European Association for palliative Care recommendations [28]
**Appropriate referral**	Referral to social prescribing	NICE [4], NCCMH Dementia care pathway [12], NHS Good Care Planning [13], SIGN [15]	New Zealand framework [17]
			Australian clinical practice guidance [16]
			World Alzheimer's report [2] (cognitive interventions)
	Referral for recommended non‐pharmacological dementia therapies	NICE [4], NCCMH Dementia care pathway [12], NHS Good Care Planning [13], SIGN [15]	New Zealand framework [17]
			Australian clinical practice guidance [16]
			World Alzheimer's report [2] (cognitive interventions)
**Other recommendations**	Carer review	NICE [4], NCCMH Dementia care pathway [12], NHS Good Care Planning [13], SIGN [15]	New Zealand framework [17]
			Australian clinical practice guidance [16]
			World Alzheimer's report [2]
	Assessment of non‐cognitive symptoms and conditions	NICE [4], NCCMH Dementia care pathway [12], NHS Good Care Planning [13], SIGN [15]	New Zealand framework [17]
			Australian clinical practice guidance [16]
			World Alzheimer's report [2]

^a^ Incentivised financially in UK primary care.

We focussed on ‘prescribing’ and ‘care planning’ quality and safety indicators. Personalised care plans and regular reviews are an evidence‐based primary care quality indicator for people with dementia [4, 6, 15]. Annual reviews are the only care process for people with dementia included in the UK quality and outcomes framework [14]. Prescribing indicators which are particularly relevant to people with dementia were selected based on synthesis of guidance [4, 12, 13, 15, 16, 17] and risk of harm [19, 20, 21, 29, 30, 31].

### 2.2 Measures of Socio‐Economic Status

SES reflects an individual's relative position within a social hierarchy, and their subsequent ability to access resources, such as healthcare [32]. Heterogenous SES measures are often used in research and clinical contexts [32, 33, 34] including composite, area‐level measures like Index of Multiple Deprivation (IMD) [35], or Townsend Quintile [36]. In studies of inequalities in older people, SES is usually operationalised by a proxy, individual‐level measure of education level, social class, or income [37]. Different measures may be more useful to answer specific research questions [37]. Table 2 details SES indicators eligible for inclusion.

**TABLE 2:** Eligible indicators of SES.

Eligible indicators of SES	
Area level measures	
Measure	Description
Index of multiple deprivation	Composite measure of 7 domains (income, employment, education, health, crime, barriers to housing and services, living environment) [38]
Townsend quintile	Composite measure of 4 domains (unemployment as a percentage of those aged 16 and over and economically active, non‐care ownership as a percentage of households, non‐home‐ownership as a percentage of households, household overcrowding as a percentage of households) [39]
Nationally derived, or study specific composite measure of socio‐economic status	Area‐level measure combing different domains, such as Index of Relative Socio‐economic Advantage and Disadvantage [40] (Australia)
Geographical area of significant deprivation described and justified in paper.	Geographical region described in relation to SES or income levels.

Individual level measures	
Measure	Description
Income level	Measured continuously or grouped, may be household, personal, current or previous.
Occupation	Measured on national scale [105], for example, occupational class.
Education level	Measured in years of education or levels (e.g., high school, more than high school).
Social class	Measured or described in relation to national scale [105]
Low‐income subsidy/eligible for financial assistance to pay for medical care (e.g., Medicaid, income assistance)	Marker of low income based on USA federal poverty guidelines.

Area‐level measures of SES are used to approximate when individual level data are not available. Evidence suggests people with high individual SES tend to live in higher SES areas [34], but area‐level measures are still only an approximation and may misclassify people based on where they live, rather than their individual SES [32, 34]. Individual level measures equally have limitations, for example someone may be highly educated but have low income, although ecological studies have found fair agreement between different SES characteristics [32, 34].

### 2.3 Search Strategy

A search of English Language literature was conducted in January 2024. Medline (All), Embase, PsychInfo and Cochrane Databases were searched. Keywords for dementia, inequality and socio‐economic deprivation were combined with indicators of high‐quality, safe primary care (Table 1). Limits were placed to include studies with human participants published from 2006 onwards.^1^ Table S1 shows the search strategy.

Grey literature was searched using terms for ‘dementia’ and ‘inequality’ in worldcat.org and clinicaltrials.gov. Further potentially relevant studies were identified through reference searching of all eligible studies, and all identified reviews. 10% abstracts were dual screened (CM/RT/KD) with 94% agreement (*ƙ* = 0.94). As ‘*ƙ*’ was > 0.8, a single reviewer screened remaining abstracts [41]. All full texts were read by one reviewer (CM), with 10% dual screened (RT); for this stage, *ƙ* = 0.91. Disagreement was resolved with a third reviewer (TB).

### 2.4 Inclusion and Exclusion Criteria

Table 3 details inclusion criteria and exclusion criteria, guided by the ‘population, concept, context’ (PCC) Prisma‐SCR framework [24].

**TABLE 3:** Inclusion and exclusion criteria.

Inclusion criteria	Exclusion criteria
**Population:** People with dementia **Concept:** **Explored variation with marker of SES for an indicator of high‐quality, safe primary care:** Annual review Personalised care plan Continuity Anti‐dementia medication prescribing Minimisation of potentially inappropriate prescribing of: anti‐cholinergic medications, anxiolytics, hypnotics and Z‐drugs Avoidance of Anti‐psychotic prescribing Medication review Polypharmacy review/avoidance **Context:** Primary care or community setting **Study types:** Quantitative Empirical studies Peer‐reviewed	Not including or reporting separate outcomes for people with dementia Not exploring indicators of quality or safety of interest^a^ Not exploring variation with a marker of SES (detailed further in this table) Secondary or tertiary care‐based study on hospitalised patients Not in English, published pre 2006^b^, non‐human studies, non‐peer reviewed (e.g., conference abstracts, poster presentations, oral presentations) Studies on patients currently hospitalised or exploring healthcare in a hospital setting Protocol not presenting results Qualitative studies^c^

^a^ All indicators detailed in Table 1 were included at abstract screening, and excluded at full text review if they did not explore prescribing or care planning indicators for this study phase.

^b^ Studies published before 2006 were excluded as this was the year that specific dementia guidance was introduced into UK General Practice [14], Although less relevant to international studies, this date represents a time when guidance for primary care for people with dementia became more detailed.

^c^ Included at abstract screening but excluded at full text review for this study phase.

### 2.5 Data Extraction

Using a standardised data extraction form [24], data extracted were: participants, concept, context, methods, year and country, indicators examined, marker of SES, quality‐rating, and key findings. The Newcastle‐Ottawa rating scales for cohort/cross‐sectional studies were applied to give an objective quality score out of 9 (≥ 7 indicting high quality, 5–6 moderate quality, < 5 low quality) [42].

## 3 Results

**FIGURE 1:**
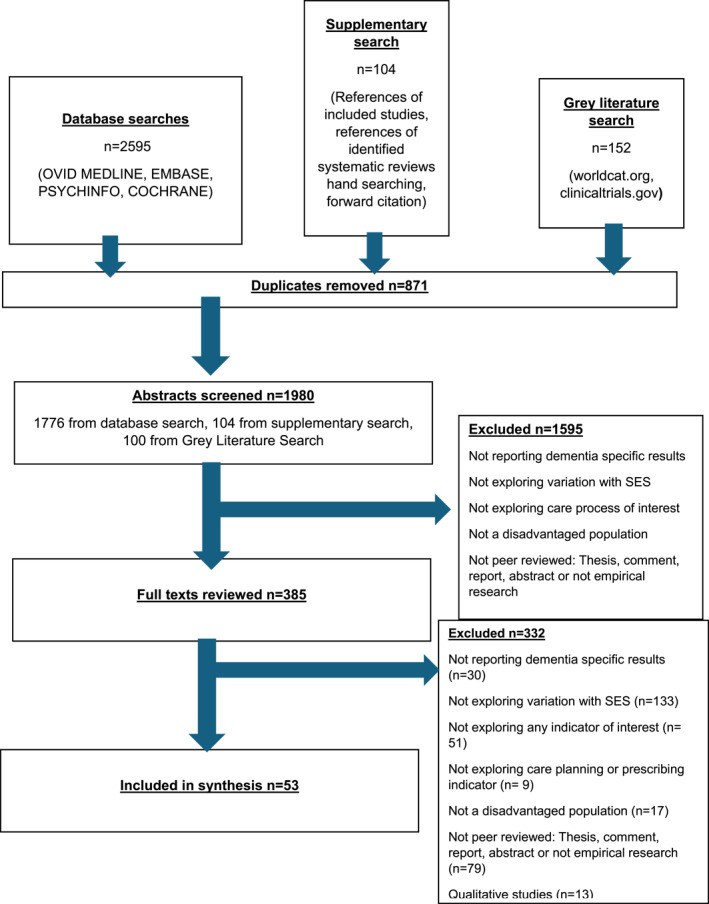
Prisma‐ScR diagram.

Fifty three studies were eligible for inclusion in the scoping review (51 primary studies and 2 reviews). Figure 1 shows the Prisma‐Scr diagram.

**FIGURE 2:**
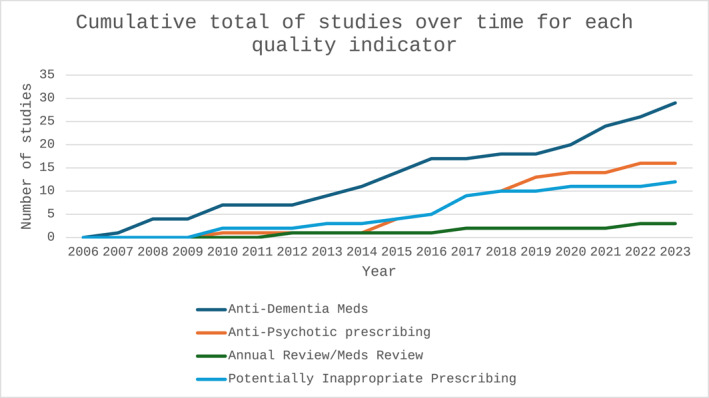
Indicators of quality and safety in included studies^a^ (^a^some studies cover more than 1 indicator).

Most included studies explored anti‐dementia medication prescribing (*n* = 29), with 16 exploring anti‐psychotic prescribing, 12 exploring PIP, 2 annual reviews and 1 exploring medication review (Figure 2); some studies explored more than one area [43, 44]. No identified studies examined how continuity‐of‐care or personalised care plans varied with SES. A wide range of SES markers were utilised. Table 4 details included studies.

**TABLE 4:** Overview of included quantitative studies.

Author	Year	Country	Data source	*N*	Indicator	Marker of SES	Key findings	Quality
Cooper [44]	2017	UK	The Health Improvement Network (THIN)	68,061	AR	Practice level Townsend Score	Deprivation was not associated with healthcare received.	High
					AP			
					PIP			
Diaz [45]	2015	Norway	Linked data from: National Population Registers	25,915	AD meds	Education	Middle or higher educated people were significantly more likely to purchase anti‐dementia medications.	High
Zilkens [46]	2014	Australia	National Pharmaceutical Benefits Scheme Database	95,274	AD meds	Australian Index of Socio‐economic disadvantage	AD medication prescribing 2.6‐fold higher in the least socioeconomic disadvantaged compared with most disadvantaged.	High
Pisu [47]	2021	USA	Random sample of USA Medicare claims database	127,512	AD meds	Deep South area versus not Deep South	Deep South beneficiaries (more deprived) were significantly more likely to have at least one anti‐dementia medication prescription.	High
Vohra [48]	2021	UK	UK National Primary care prescribing datasets.	n/a	AD meds	Deprivation level of CCG	The least deprived CCG had approximately twice the rate of prescribed anti‐dementia medications compared to the most deprived.	Moderate
Barthold [49]	2020	USA	Random 20% Medicare claims database	721,878	AD meds	Medicaid dual eligibility/low income	Higher use of Anti‐dementia meds in those in dual eligibility group, no difference for low‐income subsidy.	High
Olazaran [50]	2013	Spain	Prospective recruitment.	240	AD meds	Education	No significant differences with education level for persistence or discontinuation of AD meds	Moderate
Hoang [51]	2021	Sweden	Swedish dementia register ‐linked to insurance database	74,414	AD meds	Education/income	No association with ACHEis, but those with higher education more likely to get memantine.	High
Johnell [52]	2008	Sweden	Swedish Prescribed Drug Register	645,973	AD meds	Education	Higher education associated with significantly higher probability of AD medication, especially memantine.	High
Giebel [53]	2023	USA	United States National Alzheimer's Coordinating Center (NACC) dataset.	15,742	AD meds	Education	Education was only a significant determinant of memantine usage and was not significantly associated with other anti‐dementia medications	High
Lerner [54]	2008	USA	Cleveland Alzheimer Disease Research Center registry.	117	AD meds	Education	No significant association seen between AD medication prescribing and level of education.	Moderate
Olchanski [55]	2023	USA	Health and Retirement Study linked to Medicare Database	1299	AD meds	Education/income	Those with highest income had significantly shorter time to initiation of medications compared to those with lowest income	High
Lu [56]	2023	USA	Medicare current beneficiaries survey	1240 p	AD meds	Education/Income	Neither education nor income associated with AD medication prescribing.	High
Saleh [57]	2013	Canada	Prospective sample.	63	AD meds	Education	Those with more years of formal education were less likely to discontinue anti‐dementia medications.	Moderate
			Participants referred to memory clinic					
Lindgren [58]	2021	Sweden	Swedish Dementia Registry (SveDem) linked with tax registry	7171	AD meds	Economic position of country of origin	Foreign‐born had significantly lower odds of AD medication use and higher use of APs compared with Swedish‐born. The lower SES of the native country, the greater differences to Swedish‐born were seen.	High
Cooper [59]	2010	UK	Prospective recruitment	215	AD meds	Home ownership	Homeowners > 4 times more likely to receive AD medications compared to non‐homeowners.	Moderate
Watson [23]	2022	UK	Clinical Practice Research Datalink	142,302	AD meds	Index of multiple deprivation	Most deprived quintile with late‐onset dementia had higher rates of AD medication prescribing compared to the least deprived quintile.	High
Zuckerman [60]	2008	USA	Medicare Survey	1120	AD meds	Education/income	No association with education. AD medication users significantly less likely to live in poverty.	High
Koller [61]	2016	USA	40% sample of Medicare beneficiary database	433,559	AD meds	Low‐income subsidy	Descriptive statistics only; lower percentage receiving low‐income subsidy were prescribed AD medication	High
Thorpe [62]	2016	USA	10% sample of Medicare database enrollees	84,043	AD meds	Low‐income subsidy	Those with Medicaid low‐income subsidy had significantly lower rates of AD medications prescribed.	High
Matthews [63]	2007	UK	Medical research Council Cognitive Function Ageing Study	219	AD meds	Social class/education level	Those with higher social class, or who were more educated were significantly more likely to receive anti‐dementia medications.	High
De Moraes [64]	2018	Brasil	Brasilian Health System Dataset	16.1% dataset	AD meds	State level GDP	States with the highest GDP had the highest rates of dispensing anti‐dementia medications.	Moderate
Cooper [65]	2016	UK	THIN	77,045	AD meds	Townsend score	Least deprived quintile were 25% more likely to be initiated on anti‐dementia medications.	High
Amuah [66]	2010	USA	Saskatchewan administrative health database.	1080	AD meds	Receipt of income assistance	Discontinuation of anti‐dementia medications was significantly more likely in the least deprived group.	High
Gardette [67]	2014	12 European countries	Impact of cholinergic treatment use study dataset	557	AD meds	Education income	Education level was not significantly associated with discontinuation or switch of AD meds.	Moderate
Hernandez [68]	2010	USA	National Alzheimer's Centre Uniform dataset	3049	AD meds	Education	Higher education increased the likelihood of memantine usage.	High
Rivera‐Hernandez [69]	2022	USA	Linked data: Medicare and Nursing Home datasets	1,005,781	APs	Medicaid dual Eligibility	Those with dual eligibility were significantly more likely to receive anti‐psychotics.	High
Bargagli [70]	2019	Italy	Linked data from multiple regional datasets	24,735	APs	Area‐level composite SES measure	Those with lower SES less likely to be prescribed atypical APs.	High
Grace [71]	2018	USA	REACH study trial dataset	642	APs	Income education occupation (caregiver)	Those with a carer with higher income were more likely to receive anxiolytic medications.	High
					PIP			
Filshstein [72]	2016	USA	National Alzheimer's Co‐ordinating Centre	4741	APs	Education level	There was no difference in anti‐psychotic use for those with higher or lower levels of education.	High
Stocks [73]	2017	UK	CRPD	111,346	APs	IMD (practice level)	Antipsychotic prescribing was not associated with practice level deprivation.	High
Sivananthan [43]	2015	Canada	5 regional administrative health databases	7045	APs	Income	Those with highest income category had higher odds of AD meds and lower odds of anti‐psychotics.	High
					AD meds			
Lind [74]	2019	Australia	Electronic health record database from residential care provider	5825	APs	IRSAD area level composite	Deprivation based on IRSAD score was not related to anti‐psychotic prescribing in the multivariate model.	High
Elyn [75]	2022	France	Subsample of French National Dementia Database cohort	108,753	APs	FDEP99 composite score	Deprivation was associated with unfavourable health use in community dwelling people with dementia, but not those in a nursing home.	High
					AD meds			
Guthrie [76]	2010	UK	315 Scottish General Practices	10,058	APs	Carstairs Quintile	Most deprived quintiles were significantly more likely to be prescribed APs for >16 weeks.	High
Mar [77]	2019	Spain	Basque Health Service Database	29,864	APs	Deprivation Index	No association seen between deprivation index and anti‐psychotic prescribing.	High
Xiong [78]	2015	USA	National Alzheimer's Centre Co‐ordinating Database	8919	APs	Education	Fewer years of education was associated with increased odds of receiving an anti‐psychotic prescription.	High
Wastesson [79]	2015	Sweden	Linkage of: Swedish registries	641,566	APs	Education	Lower education level was associated with higher anti‐psychotic use.	High
Tifratene [80]	2017	France	French National Alzheimer Database	199,549	APs	Education	Higher education was protective against anti‐psychotic prescribing	High
Jones [81]	2020	UK	The Health Improvement Network	53,718	APs	Townsend deprivation score	People from more deprived areas significantly less likely to receive AD meds. No difference in chance of receiving APs or PIP	High
					AD meds			
					PIP			
Browning [82]	2022	USA	Medicare Claims Database	n/a	Medication	Gelberg‐Andersen model	Those from higher income/more educated counties more likely to enrol in medication management program.	High
					Review			
Lau [83]	2010	USA	National Alzheimer's Centre Uniform Dataset	2665	PIP	Education	In univariate analysis: Lower education level associated with increased chance of PIP. Not seen in multivariate analysis.	High
Oesterhus [84]	2017	Norway	DemWest Norwegian cohort	251	PIP	Education	Years of education not associated with rates of PIP	Moderate
					APs			
Montastruc [85]	2013	France	REAL.FR prospective cohort	684	PIP	Income education	No significant associations seen.	Moderate
Niznik [86]	2017	USA	Medicare Claims Database	4730	PIP	Low‐income subsidy	Low‐income subsidy recipients more likely to have higher anti‐cholinergic burden meds prescribed.	High
Chatterjee [87]	2010	USA	US National Nursing Home Survey data	50,993	PIP	Medicaid eligible	Those with medicaid eligibility more likely to be prescribed anti‐cholinergics.	High
Bae‐Shaaw [88]	2023	USA	Medicare Claims Database	1.6 million person years	PIP	Low income subsidy dual eligibility	Low‐income subsidy or dual eligibility recipients significantly more likely to receive 1 or more PIM	High
Cross [89]	2016	Australia	Prospective Research In MEmory clinics database	964	PIP	Education level	Education level significantly associated with PIP, but not with receiving an ACB‐3 scoring drug.	Moderate
Hyttinen [90]	2017	Finland	MEDALZ cohort database	70,718	PIP	Occupation	Socio‐economic status was not associated with PIP.	High
Connolly [6]	2012	UK	Review of primary care records in 52 general practices.	52 practices	AR	Practice level deprivation	Practice‐level deprivation was not linked to rate of annual reviews or quality of care.	High
Hanlon [91]	2015	USA	Linked data from 3 USA database sources.	1303	PIP	Education level	No association between education and PIP	High
					AD meds			

Abbreviations: AD meds = anti‐dementia medication, APs = Antipsychotic Prescribing, AR = annual review, *N* = number of participants, PIP = Potentially inappropriate prescribing.

### 3.1 Narrative Synthesis

Quantitative synthesis of results was precluded by heterogeneity between studies. Included studies used different SES measures; there was even heterogeneity within the ‘same’ SES marker. For example, ‘education’: some studies used years of education (continuous), others a binary measure. There was clinical heterogeneity between studies, some exploring all‐cause dementia and some subtypes, or exploring whether drugs were ever prescribed, duration, or discontinuation; different studies controlled for different confounders. Results are presented in a narrative synthesis.

#### 3.1.1 Anti‐Dementia Medication

‘Anti‐dementia’ medication refers to 4 medications licenced to treat dementia: donepezil, rivastigmine, galantamine (acetylcholinesterase inhibitors [AChEIs]) and memantine (NMDA‐receptor partial antagonist). These are often initiated by specialists with longer‐term prescribing in primary care. They are indicated for Alzheimer's Disease and Lewy‐Body dementia, but **not** vascular dementia [2, 4, 15]. They are recommended in evidence‐based clinical guidelines [4, 15], clinically effective when initiated correctly [2, 15], and cost‐effective [4].

Most, but not all, studies exploring anti‐dementia medication prescribing found that those with a marker of socio‐economic disadvantage were significantly less likely to receive anti‐dementia medications [43, 45, 46, 48, 51, 52, 53, 55, 57, 58, 59, 60, 61, 62, 63, 64, 65, 68, 75, 81]; 20/29 (69%) studies found a marker of lower SES was related to lower chance of receiving anti‐dementia medication. Disparities were evident across different countries [51, 53, 57, 65], and systems [51, 53, 59, 60, 61, 65] over a long timeframe (2007/8 through 2023 [52, 53, 55, 63, 92]). Studies explored different aspects of prescribing, including receiving at least one prescription [47, 49], current prescription [59], rates of prescribing [46, 48], and rates of anti‐dementia medication initiation [65]. The most used SES marker was educational level or education level with income (*n* = 13). Of those exploring education, most [45, 51, 52, 53, 55, 57, 63, 68] but not all [54, 56, 60, 67, 91] found disparities. Two studies [51, 53] found that higher education was associated with significantly increased likelihood of receiving memantine, but not AChEIs. Disparities in prescribing were seen in studies exploring variation with income [43, 61, 62], home‐ownership [59] and Townsend Quintile [65]. The heterogeneity of studies finding inequity suggests this is a robust finding across multiple SES indicators.

Both review articles explored anti‐dementia medication prescribing and variation with multiple factors of disadvantage, not just SES [23, 93]. A non‐systematic review explored patient and system factors associated with persistence and discontinuation of anti‐dementia medications [93]. The narrative conclusions discussed evidence of inequity with SES. A more comprehensive systematic review explored variation with protected characteristics for post‐diagnostic care pathways for people with dementia but did not focus on guideline recommended primary care [23].

#### 3.1.2 Anti‐Psychotic Prescribing

Despite life‐threatening risks [19] and multiple warnings to avoid their use [2, 4, 31] rates of anti‐psychotic prescribing remain high [19, 94] with evidence they are more likely to be prescribed to people with markers of lower SES [94, 95].

Sixteen studies explored variation of anti‐psychotic prescribing with a marker of SES [43, 44, 69, 70, 71, 72, 73, 74, 75, 76, 77, 78, 80, 81, 84]. Of these most (*n* = 10, 63%), but not all, found lower SES was associated with greater risk of being prescribed anti‐psychotic medications [43, 69, 70, 71, 75, 76, 78, 79, 80, 81]. The remaining six studies found no significant difference [44, 72, 73, 74, 77, 84]. Multiple markers of SES were used, covering different countries over a long timeframe (2010 – 2022) [75, 76]; the association was evident in different populations. Similarly, however, studies finding no disparities also covered a range of countries, timeframes and SES markers [44, 72, 73, 74]. Two of the studies finding no differences were high‐quality, using large, representative population‐based datasets, adjusting for multiple potential confounders [44, 73]. Neither found significant differences in rates of anti‐psychotic prescriptions based on individual or practice‐level deprivation scores [59, 73].

#### 3.1.3 Potentially Inappropriate Prescribing

The concept of ‘potentially inappropriate prescribing’ (PIP) is broad with no single definition. PIP may refer to avoiding medications likely to cause adverse effects, or sub‐optimal prescribing of indicated medications (e.g., statins). This study focussed on PIP relevant to people with dementia. The Beer's criteria state for people with dementia or cognitive impairment, anticholinergics, benzodiazepines, and Z‐drugs should be avoided [96]. These have been linked to stroke [20], falls [97], and worsening cognition [21, 30, 98]. There is evidence from the general population that people from areas of deprivation are prescribed more medications with higher cholinergic burden [29] and more anxiolytics [99].

Twelve studies focussed on PIP other than anti‐psychotics [44, 71, 81, 83, 84, 85, 86, 87, 88, 89, 90, 91]. These included anxiolytic and hypnotic medications [44, 71, 81, 88, 89, 90, 91], anti‐cholinergics [81, 83, 86, 88, 90], and other PIP against set criteria [83, 88, 90, 91]. One study classed underuse of ACHeIs as PIP, but did not analyse dementia subtypes [91]. There was less consistent evidence of disparity; 6/12 studies (50%) found those with lower SES were more likely to experience PIP [71, 83, 86, 87, 88, 89]. Again, multiple measures of SES were used in the included studies, including education level [83], low income subsidy [86, 88], Townsend score [44, 81], among others. All of those using low‐income subsidy as a marker of low SES found evidence of inequalities [86, 87, 88]. Two high‐quality studies using a large, national database found no inequalities in prescribing of anxiolytics or hypnotics with the Townsend deprivation score [44, 81].

#### 3.1.4 Annual Review and Care Planning

Annual reviews, care planning, and relational continuity of care are recommended in international guidance. There is no single guideline detailing a ‘high‐quality’ care plan/review for someone living with dementia.

Only 2 studies were identified which explored how the provision of annual reviews, dementia personalised care plans or continuity, varied with SES. Both were conducted in the UK and focussed on annual reviews. There was large variation in rates of annual reviews, ranging from 50% [44] to 80% [6]. There was no evidence from outside the UK, despite regular reviews being recommended internationally [15, 16, 17].

A cross‐sectional review of medical records, explored how rates of annual review and quality of dementia primary care varied with patient and practice level factors, including practice‐level deprivation [6]. Practice‐level deprivation was not significantly associated with rates of dementia reviews, or quality of care. This study also explored a composite score of the quality of primary care provided. Caution must be taken as the score was not validated, but overall, the study quality was high with adjustment for multiple patient and practice level factors. A key finding was that rates of annual review were high (80%), but that the quality was suboptimal in a high proportion: 26% were prescribed anti‐psychotics. This finding is supported by a large qualitative study exploring the annual review, which found variable quality, with some people not even aware reviews had taken place [22]. A high‐quality observational study using a large, national database which did not find any association between rates of annual review and practice‐level deprivation [44]. Both studies exploring annual review used area‐level SES measures, which may have limited findings.

## 4 Discussion

### 4.1 Summary of Results

Most, but not all, identified studies found evidence of disparities with SES in the quality and safety of primary care for people with dementia. Included studies primarily explored anti‐dementia medication prescribing, anti‐psychotic prescribing, and PIP; only three studies explored annual review or medication review. Most studies found inequalities in anti‐dementia medication and anti‐psychotic prescribing contrasting with studies exploring annual review, and half of those which explored PIP, which found no evidence of inequalities.

Thirty studies used large, national databases [23, 43, 44, 45, 46, 47, 48, 52, 55, 56, 60, 61, 62, 64, 65, 69, 70, 73, 74, 75, 77, 79, 81, 82, 86, 87, 88, 91]. Of these 20 (66%) found lower SES was related to poorer‐quality, less safe care. These explored anti‐dementia medication prescribing, annual reviews, PIP, and anti‐psychotic prescribing. Eleven used national dementia registries [51, 53, 54, 58, 68, 72, 78, 80, 84, 90], with 7 (64%) finding evidence of inequality; these only explored prescribing indicators. These registries were not necessarily generalisable to all people with dementia, and considering inequalities in diagnosis, may underestimate variation with SES. The remaining 10 studies used study specific or prospectively sampled cohorts [6, 50, 57, 59, 63, 67, 71, 76, 89] with 6 finding inequalities with SES. Similarly, these cohorts may have been less generalisable, more susceptible to selection bias, and underestimate inequalities through exclusion of more vulnerable patients not involved in prospective research.

Included studies generally represented low‐level, observational evidence, of varying quality; causality could not be inferred. The main limitations of included studies were the use of single, proxy markers of SES, which although appropriate, may not be fully representative. Adjustment for covariates varied between studies, with some not controlling for co‐morbidities which may have limited findings.

### 4.2 Interpretation of Results

Most identified studies explored anti‐dementia medication prescribing. A limitation of many included studies exploring AChEi prescribing is they didn't explore prescribing by dementia subtype [23, 43, 45, 47, 48, 49, 52, 53, 56, 57, 61, 62, 63]. This is important, because many cardiovascular risk factors cluster around deprivation, with higher rates of vascular disease in poorer populations – this means rates of vascular dementia may well be higher in this population [65]. ACHeIs are *not* indicated in vascular dementia. As such, it may be that people from deprived areas were **appropriately not** prescribed AChEis, if vascular dementia predominates within this group. In support of this hypothesis this, one study found that home‐owners (proxy for less socio‐economically deprived) were less frequently diagnosed with vascular dementia than renters [59]. As many of the included studies did not explore prescribing by subtype, it is difficult to interpret whether these findings reflect appropriate non‐prescribing in populations with lower SES with vascular dementia, or inequalities in provision of guideline recommended healthcare.

There were four studies, with contrasting results which found that those with lower SES were **more** likely to receive anti‐dementia medication [23, 47, 49, 50]. These explored a range of SES measures and all 4 were population database studies [23, 47, 49, 50]. A large Clinical Practice Research Datalink study found those with late‐onset dementia from the most deprived quintile were 22% **more** likely to be prescribed anti‐dementia medications, than those in the least deprived quintile [23]. This unexpected finding was hypothesised to be due to differences in health‐seeking behaviours; this study did not account for dementia subtype, which may also have influenced this finding if people with vascular dementia from lower IMD quintiles were appropriately not prescribed anti‐dementia medications. One study explored discontinuation of ACHeIs finding those least deprived were significantly more likely to discontinue anti‐dementia medications [66]. This is difficult to interpret; it may be that stopping ineffective medication represented high‐quality care. Of these 4 studies, 3 did not explore dementia subtypes, representing an important limitation [23, 47, 49].

One suggested reason for observed disparities seen in anti‐psychotic prescribing is that people with lower SES, particularly if measured using educational status, are more likely to develop behavioural and psychological symptoms of dementia (BPSD) for which anti‐psychotics are potentially indicated [79]. This would be supported by evidence of lower educational attainment being an identified risk factor for dementia, and how dementia risk factors cluster around deprivation [11]. Those with multiple risk‐factors may develop dementia younger and as such may have more severe symptoms if they live longer with the condition. They therefore have a greater chance of developing BPSD, and being prescribed anti‐psychotic medications. There is inconsistent evidence for this.

Reasons for inconsistent findings of inequity with PIP are multifactorial. Some studies suggest it is simply due to different definitions of SES [90], but the real picture is more complex. It is possible primary‐care clinicians have greater knowledge about avoiding anti‐cholinergics, anxiolytics, and z‐drugs in people with dementia compared to anti‐dementia medications; there is qualitative evidence suggesting low confidence and knowledge among primary healthcare professionals about dementia specific treatments [100, 101, 102, 103]. It is possible inequalities seen in anti‐psychotic prescribing and anti‐dementia medication prescribing reflect inequalities in *secondary care*, as specialists initiate these medications in many countries; in contrast anti‐cholinergic or anxiolytic/hypnotics may be more commonly initiated in primary care settings.

Included studies hypothesised as to why disparities were seen in prescribing indicators. Some suggested those with higher SES were better able to negotiate health systems and request medications or challenge PIP [52, 59, 65], have better communication skills [52], or that clinicians prescribing may erroneously believe those from higher SES have greater medication adherence [59]. The relationship between education and memantine was hypothesised to be due to higher educated elderly being more likely to use newer drugs [51, 52, 53]. It is likely a combination of factors led to observed inequity. Clinician factors must be considered; many lower SES areas are under‐resourced [8], which may mean people living in these areas have less access to a clinician specialised in dementia.

It is difficult to draw firm conclusions about how annual reviews and care planning for people with dementia vary with SES from the 2 identified studies. It is notable that both found no differences with deprivation (in contrast to prescribing indicators), and both were conducted in UK populations. The annual review for people with dementia is financially incentivised through QOF in the UK; this may have influenced rates of reviews, but not necessarily quality. Larger studies are required to understand better this.

### 4.3 Strengths and Limitations

To our knowledge, this is the first scoping review exploring guideline recommended indicators of quality and safety of primary care provided for people with dementia. Studies exploring variation with a range of SES indicators were included. This allowed a greater number of studies for inclusion and a clearer picture of existing inequities to be formed, increasing robustness and generalisability. The results are presented to map the existing literature, which shows clear and important gaps, particularly for care planning/reviews; this is important given these are recommended in international guidance. All included studies were graded moderate or high quality using the Newcastle‐Ottawa Scale.

We cannot be certain all relevant studies were identified. For example, for PIP, all possible individual drug names were not searched. However, given the wide search strategy, and reference searching of included studies, missed studies are likely to be minimal. Quantitative synthesis of results was not undertaken due to the heterogenous nature of included studies, findings from high‐quality studies with rigorous methodology and attempts to reduce bias were included alongside studies with methodological limitations. 10% abstracts and full‐texts were dual screened, with high agreement, however dual screening at every stage is recommended best practice [24];single reviewer screening may have increased the risk of missing relevant studies and bias. Nevertheless, successful single reviewer screening is used in review methodologies [104], especially where *ƙ* > 0.8 for dual‐screened studies. Studies including individual and area‐level measures of SES were included; area‐level measures may have misclassified some people. Including only English Language studies means studies from LMICs may have been excluded, this is important given the projected increases in dementia prevalence in LMICs; further work including studies in languages other than English is needed. Finally, given the narrative synthesis of findings, nuances of included studies may have been lost, for example, findings in a specific population; Table 4 aimed to mitigate this.

### 4.4 Implications for Practice and Future Research

Clinicians should be aware of disparities in the quality and safety of primary care for people with dementia. Disparity was evident over a range of indicators and markers of SES. Of particular concern are the findings related to anti‐psychotic drug prescribing. Despite multiple global warnings to reduce the use of anti‐psychotic medications in people with dementia, except in extreme circumstances, these medications remain frequently used, with evidence suggesting more frequent use in people lower SES. Further work exploring inequality in anti‐dementia medication prescribing in dementia subtypes is urgently needed, particularly focussing on if dementia subtype varies with deprivation.

Only one identified study [64] explored inequalities in a LMIC. Although this may reflect the English language limit, given projected increases in dementia prevalence in LMICs in coming years, this represents a vital area for future research.

The scoping review found most studies to date explored prescribing indicators, with far fewer focusing on non‐pharmacological aspects of care like care planning and/or reviews, despite guidance suggesting these represent high‐quality primary care for people with dementia. Exploring the quality of annual health reviews and the process of care planning, and if/how this varies with SES is an important area for future research.

## 5 Conclusion

The scoping review found evidence of inequalities in the quality and safety of primary care for people with dementia particularly in anti‐dementia medication and anti‐psychotic prescribing. Far fewer studies were identified exploring care planning/reviews for people with dementia. Literature to date has shown inequalities in primary care for people dementia with SES; what this scoping review adds is confirmation of the breadth and diversity of these inequalities in terms of prescribing indicators. Furthermore, we identify important gaps in the existing literature, identifying priority areas for future research. Clinicians and researchers need to be aware of these existing inequalities in primary care for people with dementia; tackling these should become a priority area for clinical practice, research, and policy‐making.

## Author Contributions

C.M. designed the study with support from T.B., D.M.A., E.K., and L.R. C.M. drafted the manuscript. K.D. and R.T. acted as second reviewers of abstracts and full‐texts, T.B. acted as third reviewer. T.B., D.M.A., L.R., K.D., R.T. and E.K. critically revised the manuscript. C.M. is the guarantor of this work and, as such takes responsibility for the integrity of the data and the accuracy of analysis.

## Ethics Statement

The authors have nothing to report.

## Consent

The authors have nothing to report.

## Conflicts of Interest

The authors declare no conflicts of interest.

## Permission to Reproduce Material From Other Sources

The authors have nothing to report.

## Supporting information

Table S1

## Data Availability

No additional data are available.

## Endnotes

^1^
Studies published before 2006 were excluded as this was the year that specific dementia guidance was introduced into UK General Practice [14], Although less relevant to international studies, this date represents a time when guidance for primary care for people with dementia became more detailed.
