# Supplementary material for: Inequalities in the Quality and Safety of Post‐Diagnostic Primary Care for People With Dementia: A Scoping Review
Source: Int J Geriatr Psychiatry. 2024 Dec 23;39(12):e70035. doi: 10.1002/gps.70035 (PMC11666866; doi:10.1002/gps.70035)
Supplement: Supplementary file 1 — Table S1 [file GPS-39-e70035-s001.docx]

**Supplementary table 1: database search terms**

| **Population** | **AND** | **Exposure/concept** | **Comparator** | **AND** | **Outcome** |
| --- | --- | --- | --- | --- | --- |
| People with dementia |  | Socio-economic factors | Affluence |  | Primary care processes |
| ‘Dementia’ OR ‘Alzheim*’ OR ‘Lewy-Body’ OR ‘Frontotemporal’ OR ‘Demen*’ |  | ‘socio-economic’ OR ‘ineq*’ OR ‘depriv*’ OR ‘dispar*’ OR ‘ethn*’ | [‘ineq*’ OR ‘depriv*’ OR ‘dispar*’] |  | **Prescribing:** ‘anti-dementia medication’ OR ‘acetylcholinesterase inhibitor’ OR ‘memantine’ OR ‘donepezil’ OR ‘Aricept’ OR ‘galantamine’ OR ‘rivastigmine’ OR ‘anti-psychotic’ OR ‘neuroleptic’  ‘Polypharmacy’ OR ‘potentially inappropriate prescribing’ OR ‘PIP’ OR ‘anti-cholinergic’ OR ‘medication review’ OR ‘anxiolytic’ OR ‘hypnotic’ OR ‘z-drugs’  **Care planning/review: ‘c**are planning’ OR ‘care plan’ OR ‘annual review’ OR ‘continuity’ OR ‘continuity of patient care’ OR ‘missed appointments’ OR ‘frequency of appointment’ or ‘appointment type’  **Referral** – ‘social prescribing’ OR ‘referral’ OR ‘cognitive rehabilitation’ OR ‘group reminiscence’  **End-of-life care** – ‘advance care planning’ OR ‘palliative care’ OR ‘place of death’ OR ‘end of life care’ |
